# Most Effective Interventions for Improving Upper Extremity Function in Patients with Hemiparesis

**DOI:** 10.26502/fccm.92920475

**Published:** 2025-12-24

**Authors:** Andre Aabedi, Daniel Mashiach, Marcel P. Fraix, Devendra K. Agrawal

**Affiliations:** 1 Departments of Translational Research, College of Osteopathic Medicine of the Pacific, Western University of Health Sciences, Pomona, California 91766 USA; 2 Physical Medicine and Rehabilitation, College of Osteopathic Medicine of the Pacific, Western University of Health Sciences, Pomona, California 91766 USA

**Keywords:** Constraint-Induced Movement Therapy (CIMT), Hemiparesis, Individualized Treatment, Multimodal Rehabilitation, Neuromodulation, Neuromuscular Electrical Stimulation (NMES), Neuroplasticity, Rehabilitation, Robotics, Robotic-assisted rehabilitation, Task-specific training, Upper Extremity Function

## Abstract

Hemiparesis, commonly resulting from stroke, leads to significant impairments in upper extremity function, limiting daily activities and reducing quality of life. Effective rehabilitation strategies are essential to enhance motor recovery and restore functional independence. This review evaluates the most effective interventions for improving upper extremity function in patients with hemiparesis. A comprehensive literature review was conducted, analyzing systematic reviews, randomized controlled trials, and clinical guidelines. The efficacy of various interventions, including task-specific training, constraint-induced movement therapy (CIMT), neuromuscular electrical stimulation (NMES), mirror therapy, virtual reality, bilateral arm training, pharmacological approaches, and robotic-assisted rehabilitation, was assessed based on their impact on motor function and daily activities. The review highlights the role of neuroplasticity in motor recovery, emphasizing interventions that promote cortical reorganization. Task-specific training, CIMT, and NMES demonstrate strong evidence in enhancing motor function. Emerging technologies, such as brain-computer interfaces and robotics, show promise in optimizing rehabilitation outcomes. Factors influencing recovery, including stroke severity, time since onset, and patient motivation, are discussed. Studies consistently support the effectiveness of CIMT and task-specific training in improving upper extremity function. NMES and mirror therapy are beneficial adjunct therapies, particularly for patients with moderate impairment. Virtual reality and robotics enhance engagement and motor learning, while pharmacological and stem cell therapies are emerging areas with potential but require further research. A multimodal rehabilitation approach combining task-oriented therapies, neuromodulation, and emerging technologies yields the best outcomes for upper extremity recovery in hemiparesis patients. Future research should focus on optimizing individualized treatment plans and integrating novel therapeutic modalities to maximize functional gains.

## Introduction

Hemiparesis, a common consequence of stroke, refers to the weakness or partial paralysis affecting one side of the body. This condition significantly impacts upper extremity function, leading to difficulties in performing activities of daily living and reducing overall quality of life. The American Heart Association and American Stroke Association highlight that upper extremity impairments are prevalent among stroke survivors, with many experiencing persistent deficits that hinder their independence and participation in daily activities [1]. The impairment in upper extremity function due to hemiparesis can manifest as reduced strength, coordination, and dexterity, making tasks such as dressing, eating, and personal hygiene challenging. This can lead to increased dependency on caregivers and, in severe cases, may contribute to institutionalization. Effective rehabilitation strategies are crucial to maximize recovery and improve functional outcomes for these patients [1,2].

Rehabilitation is crucial in restoring motor function in patients with hemiparesis, particularly following a stroke. The American Heart Association/American Stroke Association emphasizes the importance of task-specific training, which involves repeated, challenging practice of functional, goal-oriented activities. This approach is foundational in interventions such as constraint-induced movement therapy and neuromuscular electrical stimulation, which have shown efficacy in improving upper extremity function [1].

Task-specific training is supported by multiple systematic reviews and clinical guidelines. For instance, the U.S. Department of Veterans Affairs and the United States Department of Defense highlight the effectiveness of task-specific practice in improving motor function, gait, posture, and activities of daily living [3]. This method involves whole-task or pre-task movements, such as grasping and gripping, which are repeated multiple times during therapy sessions.

Intensive rehabilitation, including high-intensity and repetitive task-specific practice, has been shown to significantly improve motor outcomes. A study published in Stroke demonstrated that intensive arm motor therapy improved upper limb motor function in a substantial fraction of patients, indicating enhanced functional recovery [2]. Moreover, recent advancements in rehabilitation techniques, such as robot-assisted training and virtual reality, offer promising outcomes. These technology-based interventions can be tailored to individual patient needs, potentially maximizing motor function recovery [4–6]. The purpose of this paper is to identify the most effective interventions for improving upper extremity function in a patient with hemiparesis.

## Pathophysiology of Hemiparesis

**Figure 1:**
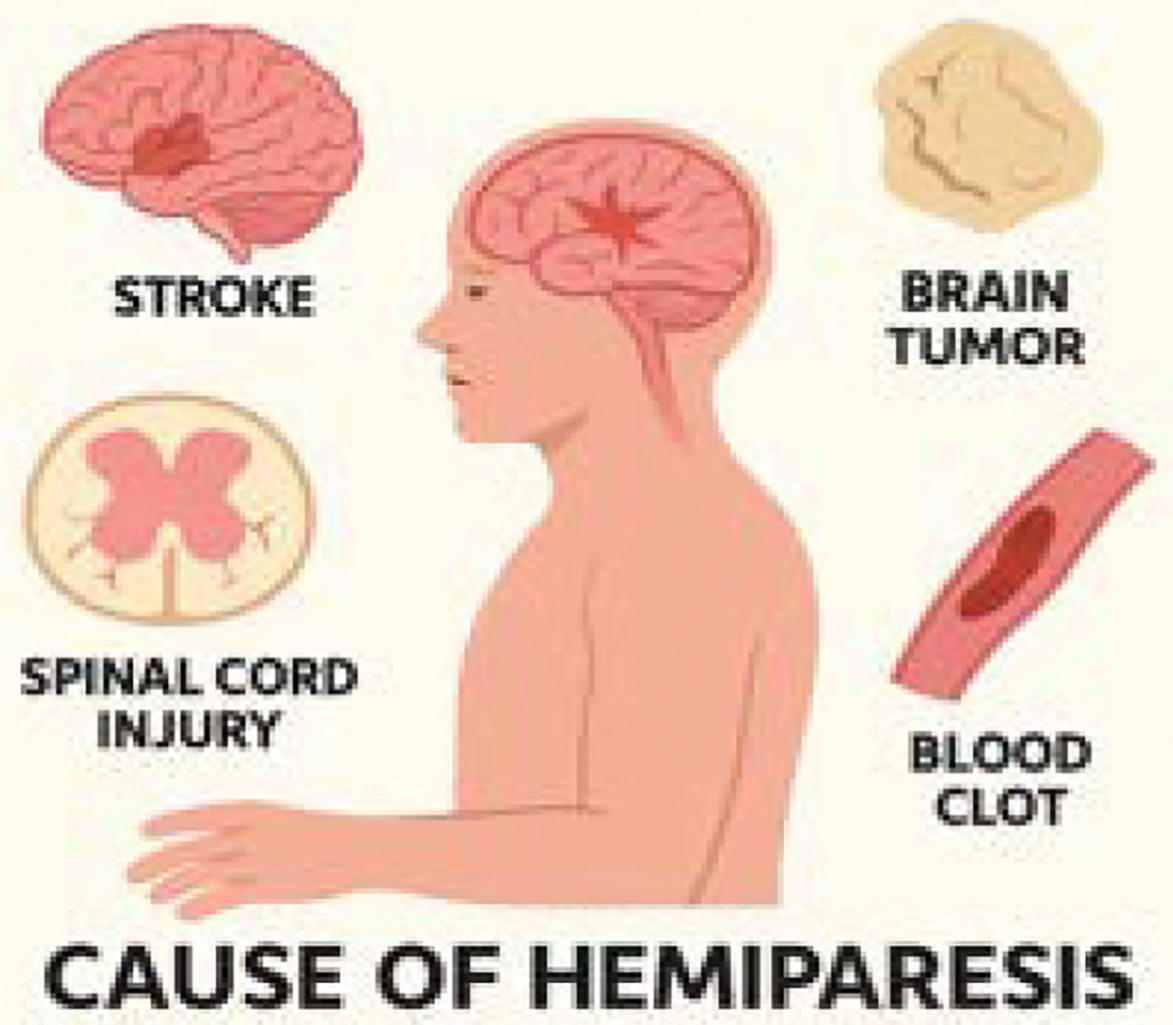
This schematic diagram illustrates the causes of hemiparesis.

The pathophysiology primarily revolves around damage to the corticospinal tract, which is crucial for voluntary motor control. This could be due to several reasons: ischemia due to the lack of blood flow to the brain causing damage to the motor cortex, hemorrhage in the brain may compress or destroy motor pathways, traumatic brain injury may directly damage the motor cortex and other parts of the brain that control motor function, and brain tumors compressing motor pathways. Lesions in the corticospinal tract, particularly at the corona radiata and internal capsule, are strongly associated with hemiparesis [3]. This damage disrupts the transmission of motor signals from the brain to the spinal cord, leading to muscle weakness or paralysis, sensory loss, spasticity, and cognitive impairment on the contralateral side of the body (Figure 1).

Additionally, the peri-Sylvian cortical and subcortical regions, including the putamen, are implicated in hemiparesis. These areas are involved in motor planning and execution, and their damage can further impair motor function [3]. The integrity of white matter tracts connecting the primary motor cortex, ventral premotor cortex, and posterior parietal motor areas is also crucial. Disruption of these pathways can significantly affect motor function post-stroke [1]. Neuroplastic changes in the brain post-stroke also play a role. There is often a loss of gamma-aminobutyric acid-mediated interhemispheric inhibition, which normally helps balance motor activity between hemispheres. This loss can lead to hyperexcitability in the contra lesional hemisphere, further complicating motor recovery [7]. Functional connectivity analyses have shown that stroke induces changes in both intra- and inter-hemispheric interactions, affecting motor networks and contributing to hemiparesis [8].

Hemiparesis involves motor impairments and functional limitations that significantly impact a patient’s quality of life. The motor impairments in hemiparesis include muscle weakness, spasticity, and abnormal muscle activation patterns [2]. These impairments are due to damage to the supraspinal centers and subsequent physiological changes in the muscles and motor units, which reduce muscle force generation and coordination [9].

Functional limitations associated with hemiparesis are extensive. For the upper extremities, patients often experience loss of dexterous movement, reduced sensation, and impaired coordination, which hinder their ability to perform daily activities such as reaching, grasping, and manipulating objects. The American Heart Association notes that these deficits can lead to learned nonuse or maladaptive use of the affected limb, further exacerbating functional limitations [1].

Neuroplasticity, the brain’s ability to reorganize itself by forming new neural connections, plays a crucial role in the recovery of upper extremity function in patients with hemiparesis following a stroke. This process is driven by both spontaneous recovery and rehabilitation-induced plasticity. Several studies have demonstrated that targeted rehabilitation can enhance neuroplasticity and improve motor function. For instance, intensive hand and finger rehabilitation activities, such as those involving virtual rehabilitation, have shown significant improvements in motor skills and cortical reorganization in patients with subacute stroke [1,10–12]. Similarly, repetitive transcranial magnetic stimulation combined with motor training has been shown to facilitate use-dependent plasticity and improve motor function in chronic stroke patients [11–13].

Bilateral movement training is another effective approach, leveraging interlimb coordination principles to promote cortical reorganization and motor recovery. This method can enhance motor cortex disinhibition and recruit ipsilateral pathways to support the damaged hemisphere [14]. Additionally, motor imagery-based brain-computer interface rehabilitation programs have been shown to enhance upper extremity performance and cortical activation, suggesting their potential utility in stroke rehabilitation [10,12].

## Conventional Interventions

Physical and occupational therapy play a crucial role in improving upper extremity function in patients with hemiparesis, particularly following a stroke. The American Heart Association and American Stroke Association recommend task-specific training, which involves practicing functional tasks that are graded to challenge individual capabilities, practiced repeatedly, and progressed in difficulty [8]. This approach is based on the principle that practice of an action results in improved performance of that action.

Activity-based task-oriented training (task-oriented training) has been shown to be effective in improving upper extremity motor function, motor performance, and activities of daily living performance in adults with stroke. A systematic review found strong evidence supporting the effectiveness of hospital-based task-oriented training and moderate evidence for home-based task-oriented training [15]. This aligns with the occupational therapy philosophy of using functional and meaningful activities in practice. The American Heart Association/American Stroke Association guidelines recommend strengthening exercises as an adjunct to functional task practice, highlighting their potential benefits in upper extremity rehabilitation [1]. This is supported by a meta-analysis which found that strength training improves grip strength and upper-limb function without increasing spasticity or pain [2].

Range of motion exercises are also crucial. They help maintain joint flexibility and prevent contractures, which are common complications in hemiparetic patients. Studies have shown that range of motion exercises, when integrated into rehabilitation programs, can improve active range of motion and functional outcomes. Constraint-Induced Movement Therapy is a well-established intervention for improving upper extremity function in patients with hemiparesis following a stroke. Constraint-Induced Movement Therapy involves restraining the less-affected limb to encourage the use of the affected limb through repetitive task practice and behavioral shaping. The EXCITE trial demonstrated that Constraint-Induced Movement Therapy significantly improves upper extremity function in stroke patients, with benefits persisting for at least one year. Patients in the Constraint-Induced Movement Therapy group showed greater improvements in the Wolf Motor Function Test and the Motor Activity Log compared to those receiving usual care [12,16,17].

A systematic review and meta-analysis by de Azevedo et al. [18] confirmed that constraint-induced movement therapy leads to superior outcomes in motor function and activities of daily living compared to conventional therapies. The review highlighted significant improvements in measures such as the Fugl-Meyer Assessment, Wolf Motor Function Test, and the Modified Barthel Index [18]. The American Heart Association/American Stroke Association guidelines recommend constraint-induced movement therapy for patients with some baseline ability to control wrist and finger extension, noting its efficacy in improving upper extremity activity, participation, and quality of life [1].

## Neuromodulatory and Technological Approaches

Functional Electrical Stimulation (FES) plays a significant role in improving upper extremity function in patients with hemiparesis following a stroke. FES involves the application of electrical currents to stimulate muscle contractions, aiding motor recovery by enhancing neuromuscular reeducation and promoting functional movements. Multiple systematic reviews and meta-analyses have demonstrated the efficacy of FES in post-stroke rehabilitation. For example, the Department of Veterans Affairs stroke rehabilitation guideline highlights that FES, NMES, and TENS are associated with statistically superior results in upper extremity motor outcomes compared to placebo or no electrical stimulation, with improvements measured by the Fugl-Meyer Assessment and other functional scales [12]. A network meta-analysis found that electrical stimulation combined with task-specific training is among the most effective interventions for upper limb motor function after stroke [2]. Another meta-analysis showed that FES therapy leads to significant improvements in arm function, as measured by the Fugl-Meyer Assessment and Action Research Arm Test, compared to placebo [19]. Furthermore, combining FES with occupational therapy yields greater improvements in Fugl-Meyer scores and quality of life than occupational therapy alone or tDCS plus occupational therapy [20].

**Figure 2:**
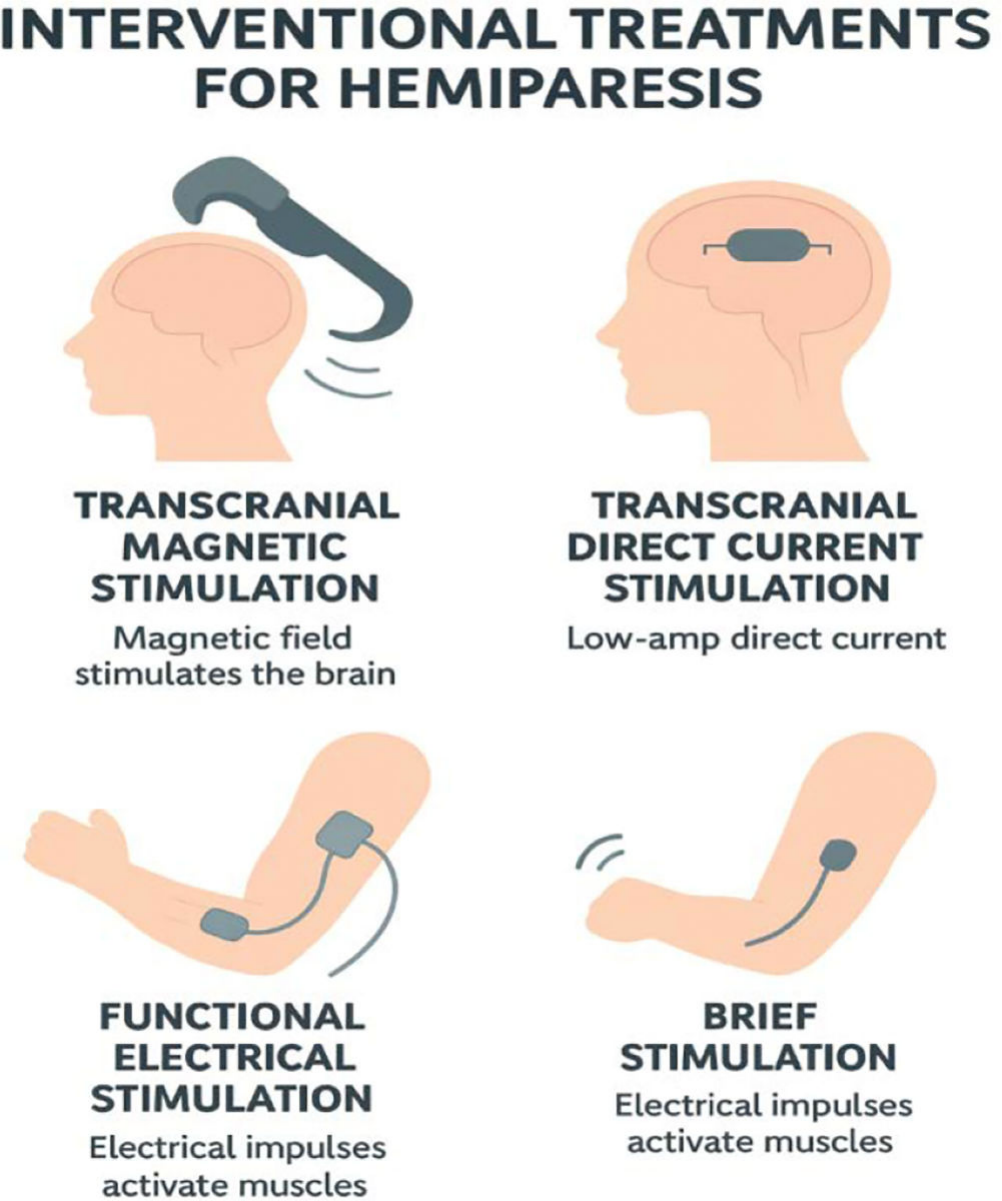
The schematic diagram illustrates different types of interventional procedures that can be used in treat upper extremity hemiparesis.

Various FES systems, including manually controlled, brain-computer interface-controlled, and electromyography-controlled FES, have demonstrated favorable outcomes for upper limb recovery (Figure 2). The VA guideline and recent meta-analyses support the use of EMG-triggered FES and contralaterally controlled FES, which show greater improvements in motor outcomes compared to conventional NMES [12]. These interventions result in significant gains in Fugl-Meyer Assessment and Action Research Arm Test scores, indicating effective enhancement of upper limb motor function [19,20].

Transcranial Magnetic Stimulation (TMS) and Transcranial Direct Current Stimulation (tDCS) are non-invasive brain stimulation techniques that have shown promise in improving upper extremity function in patients with hemiparesis following a stroke (Figure 2). Systematic reviews and network meta-analyses confirm that rTMS, especially high-frequency protocols applied to the ipsilesional motor cortex, significantly improve upper limb motor function and hand dexterity, with benefits most pronounced in the first three months post-stroke [21–24]. Among non-invasive brain stimulation protocols, anodal tDCS and high-frequency rTMS are ranked as the most effective for improving Fugl-Meyer scores and activities of daily living [24]. These findings are supported by meta-analyses showing that excitatory stimulation protocols (intermittent TBS, anodal tDCS, and HF-rTMS) are most effective for motor recovery and activities of daily living in both acute/subacute and chronic stroke [22,23].

Transcranial direct current stimulation involves applying a low electrical current to the scalp to modulate neuronal activity. Anodal transcranial direct current stimulation over the ipsilesional motor cortex can enhance motor recovery by increasing cortical excitability, while cathodal transcranial direct current stimulation over the contralesional motor cortex can reduce interhemispheric inhibition [24]. Studies have shown that both anodal and cathodal transcranial direct current stimulation can improve upper extremity function in stroke patients. For instance, a systematic review and meta-analysis by Tang et al. [25] found that transcranial direct current stimulation significantly improved upper extremity motor function and activities of daily living, with the highest efficacy observed in subacute stroke patients.

Combining transcranial direct current stimulation with other rehabilitation techniques, such as motor imagery-assisted brain-computer interface training, has also shown potential benefits. Kashoo et al. [26] demonstrated that transcranial direct current stimulation priming before motor imagery training could enhance motor function in chronic stroke patients.

## Task-Oriented and Motor Learning-Based Interventions

Mirror therapy (MT) has been shown to be an effective intervention for improving upper extremity function in patients with hemiparesis following a stroke. The therapy involves placing a mirror in the midsagittal plane of the patient, reflecting the movements of the non-paretic limb as if they were being performed by the paretic limb.

A comprehensive review by Thieme et al. [30] in the Cochrane Database of Systematic Reviews (2018) found moderate-quality evidence that mirror therapy significantly improves motor function and motor impairment in stroke patients. Specifically, the standardized mean difference (SMD) for motor function was 0.47 (95% confidence interval 0.27 to 0.67) and for motor impairment was 0.49 (95% confidence interval 0.32 to 0.66) [30]. Additionally, mirror therapy was shown to improve activities of daily living (SMD 0.48, 95% confidence interval 0.30 to 0.65) and reduce pain (SMD −0.89, 95% confidence interval −1.67 to −0.11) [30]. Further supporting evidence from a systematic review and meta-analysis by Hsieh et al. (2025) indicated that mirror therapy significantly improved motor impairment (effect size 0.473 [0.274–0.673]), motor function (0.266 [0.059–0.474]), and activities of daily living (0.461 [0.25–0.671]) in subacute stroke patients [31]. The therapy was particularly effective when administered more than five times a week over a period of four weeks [30]. In acute stroke patients, the Department of Veterans Affairs guideline (2024) also supports the use of mirror therapy, noting statistically and clinically significant benefits for motor outcomes, including motor function, motor impairment, and ADLs, with typical protocols involving three to seven sessions per week for two to eight weeks [12].

Virtual reality (VR) and gaming-based therapy have shown promise in improving upper extremity function in patients with hemiparesis, particularly post-stroke. These interventions leverage immersive environments and interactive tasks to enhance motor recovery through increased engagement and repetitive practice. The 2025 Cochrane review by Laver et al. found that virtual reality may be beneficial in slightly improving upper limb function and activity compared to alternative therapy approaches (SMD 0.20, 95% CI 0.12 to 0.28; low-certainty evidence), and greater benefits were seen when VR was used in addition to usual care (SMD 0.42, 95% CI 0.26 to 0.58; moderate-certainty evidence) [32]. Hybrid VR combined with conventional therapy is more effective than conventional therapy alone for motor function and manual dexterity, with sustained benefits over time (SMD = 0.44 for motor function, SMD = 0.33 for manual dexterity) [6]. The Department of Veterans Affairs guideline (2024) also notes statistically significant improvements in upper extremity motor function, range of motion, and muscle strength with VR-supported exercise compared to usual care or no therapy [12]. Optimal improvements are observed with hybrid sessions of 31 to 59 minutes daily [6].

A study found that immersive virtual reality-based hand rehabilitation games significantly improved hand motor functions in subacute stroke patients, with notable improvements in clinical outcome measures such as the Fugl-Meyer Assessment-Upper Extremity and the Action Research Arm Test [33,34]. This suggests that cognitive engagement and visual feedback in virtual reality can effectively enhance motor function. Additionally, a systematic review and meta-analysis by Chen et al. [5] confirmed that virtual reality-supported exercise therapy significantly improved upper extremity motor function, range of motion, and muscle strength compared to conventional therapy. However, the benefits were not consistently maintained post-intervention, indicating the need for ongoing therapy to sustain improvements [12].

Bilateral Arm Training has been shown to play a significant role in improving upper extremity function in patients with hemiparesis following a stroke. Bilateral Arm Training involves the simultaneous use of both the affected and unaffected limbs, which can facilitate neuroplasticity and motor recovery. Several systematic reviews and meta-analyses have compared the efficacy of Bilateral Arm Training with Unilateral Arm Training and conventional therapy. Chen et al. (2019) found that Bilateral Arm Training yielded superior improvements in motor impairment, as measured by the Fugl-Meyer Assessment-Upper Extremity, compared to Unilateral Arm Training [35]. However, no significant differences were observed in functional performance measures such as the Wolf Motor Function Test, Action Research Arm Test, and Box and Block Test [35].

Chen et al. (2022) also reported that Bilateral Arm Training demonstrated significantly greater improvements in motor impairments than conventional therapy, particularly in patients with mild impairments in the chronic phase of stroke [5]. The study highlighted that higher doses of Bilateral Arm Training were associated with better outcomes, and bilateral functional task training was particularly effective in improving both motor impairments and functional performance [5]. Neuroimaging studies have shown that Bilateral Arm Training can enhance activation in the ipsilesional primary motor area, supplementary motor area, and primary sensory cortex, and improve interhemispheric connectivity, which may contribute to its effectiveness in motor recovery [34].

## Pharmacological and Biologic Interventions

Medications play a significant role in improving upper extremity function in patients with hemiparesis, particularly following a stroke. However, there is currently no evidence in the medical literature supporting cyproheptadine hydrochloride as an effective intervention for upper extremity motor recovery in stroke survivors. For pharmacological interventions, the current evidence base is limited and does not support routine use for upper extremity motor recovery after stroke [13].

The American Heart Association and American Stroke Association guidelines recommend considering neuromuscular electrical stimulation (NMES) for individuals with minimal volitional movement, as it may improve upper extremity activity when combined with task-specific training [1]. Additionally, robotic therapy is suggested for delivering more intensive practice for individuals with moderate to severe upper limb paresis, although its superiority overdose-matched conventional therapy remains uncertain [10]. High-quality evidence supports electrical stimulation combined with task-specific training, high-volume constraint-induced movement therapy, and strength training as among the most effective interventions for upper limb motor function after stroke [2,12]. Constraint-induced movement therapy (CIMT), mental practice, mirror therapy, virtual reality, and repetitive task practice also have moderate-quality evidence for benefit.

Stem cell therapy and growth factors are emerging treatments that show promise in improving upper extremity function in patients with hemiparesis following a stroke. However, none of the provided references support the efficacy of stem cell therapy or growth factors for upper extremity motor recovery in stroke patients. Current guidelines and systematic reviews do not recommend these interventions outside of research settings [3,17]. NSCs, when transplanted intracerebrally, have shown promise in promoting functional recovery by forming new neurons and supporting neuroregeneration.

Growth factors, such as vascular endothelial growth factor and brain-derived neurotrophic factor, play a significant role in neuroprotection, neurogenesis, and synaptic plasticity. Combining growth factors with stem cell therapy can enhance the survival and integration of transplanted cells, thereby improving therapeutic outcomes. This combinatorial approach has been shown to stimulate endogenous neurogenesis, reduce inflammation, and provide neuroprotection, which collectively contribute to functional recovery. While these mechanisms are supported by preclinical and early-phase clinical research, current clinical guidelines and systematic reviews emphasize that the most effective, evidence-based interventions for improving upper extremity function in hemiparesis remain intensive, task-specific rehabilitation therapies, electrical stimulation, and neuromodulation approaches [13,36].

Clinical studies and reviews suggest that while early-phase trials indicate safety and potential efficacy, larger randomized controlled trials are needed to establish definitive clinical effectiveness and optimize protocols regarding cell type, dosage, timing, and administration routes. The integration of these therapies with rehabilitation protocols, such as physical and occupational therapy, may further enhance functional outcomes by promoting tissue restoration and neuroplasticity. Current evidence supports combining novel interventions such as neuromodulation and electrical stimulation, with high-dose, task-oriented rehabilitation to maximize upper limb recovery, but robust data for growth factor and stem cell therapies in clinical practice are still lacking [37,38].

## Factors Influencing Rehabilitation Outcomes

Rehabilitation outcomes for improving upper extremity function in patients with hemiparesis are influenced by several key factors. According to the American Heart Association/American Stroke Association guidelines, task-specific training, including activities of daily living and instrumental activities of daily living, is essential for upper extremity rehabilitation. This involves practicing functional tasks that are progressively challenging and repeated frequently [3,16].

Motor ability and initial severity of impairment are significant predictors of recovery. Patients with better initial motor function and cognitive abilities, as assessed by tools like the Fugl-Meyer Assessment and the Montreal Cognitive Assessment, tend to show more substantial improvements [3,6]. Additionally, the time since stroke onset is inversely related to recovery, with earlier rehabilitation initiation being more beneficial [4,6]. Psychosocial factors, including motivation and self-efficacy, also play a crucial role. Higher motivation levels are strongly linked to better motor function recovery and greater independence in daily activities [3]. The relationship between healthcare professionals and patients, along with the availability of resources, can impact engagement and adherence to rehabilitation programs [12].

## Factors Influencing Rehabilitation Outcomes

The findings from this review indicate that task-specific training, CIMT, and NMES are among the most effective interventions for improving upper extremity function in patients with hemiparesis. Mirror therapy, virtual reality, and robotics also contribute significantly to motor recovery by enhancing engagement and neuroplasticity. Additionally, pharmacological treatments and stem cell therapies show potential but require further investigation to establish their efficacy. A multimodal and individualized rehabilitation approach incorporating these interventions provides the best outcomes, emphasizing the need for personalized treatment strategies and continued research to enhance rehabilitation techniques.

## Key points:

- Hemiparesis commonly resulting from stroke, leads to significant impairments in upper extremity function, limiting daily activities and reducing quality of life.
- Rehabilitation strategies are essential to enhance motor recovery and restore functional independence.
- Task-specific training is assessed based on its impact on motor function and daily activities alongside other interventions such as constraint-induced movement therapy (CIMT), neuromuscular electrical stimulation (NMES), and mirror therapy.
- Neuroplasticity plays a crucial role in motor recovery, emphasizing interventions that promote cortical reorganization.
- CIMT (Constraint-Induced Movement Therapy) has strong evidence supporting its effectiveness in improving upper extremity function.
- NMES (Neuromuscular Electrical Stimulation) and mirror therapy are beneficial adjunct therapies, particularly for patients with moderate impairment.
- Robotics and brain-computer interfaces show promise in optimizing rehabilitation outcomes through emerging technologies.
- Pharmacological and stem cell therapies are emerging areas with potential but require further research to establish their effectiveness.
- Multimodal rehabilitation approaches that combine task-oriented therapies, neuromodulation, and emerging technologies yield the best outcomes for upper extremity recovery.
- Individualized treatment plans should be optimized through future research to integrate novel therapeutic modalities and maximize functional gains.
